# Metabolomics-Based Biomarkers for Frailty in Chinese Older Adults

**DOI:** 10.1093/geroni/igab046.2048

**Published:** 2021-12-17

**Authors:** Yiming Pan, Pan Liu, Yun Li, Lina Ma

**Affiliations:** 1 Capital Medical University Xuanwu Hospital, Beijing, Beijing, China (People's Republic); 2 Xuanwu Hospital, Capital Medical University, National Research Center for Geriatric Medicine, Beijing, Beijing, China (People's Republic)

## Abstract

Background: Frailty is a clinical state characterized by decline in physiological function, and increased vulnerability to adverse outcomes. The biological mechanisms underlying frailty have been extensively studied in recent years. Advances in the multi-omics platforms have provided new information on the molecular mechanisms of frailty. Thus, identifying omics-based biomarkers is helpful for both exploring the physiological mechanisms of frailty and evaluating the risk of frailty development and progression. Objective: To identify metabolomics biomarkers and possible pathogenic mechanisms for frailty with untargeted-metabolomics profiling. Methods: LC-MS-based untargeted metabolomics analysis was performed on serum samples of 25 frail older inpatients and 49 non-frail older controls. The metabolomics profiling was compared between the two groups. Results: We identified 349 metabolites belonging to 46 classes, in which 2 were increased and 3 were decreased in frail older adults. Citrate cycle (with up-regulated cis-Aconitic acid, Fumaric acid, L-Malic acid, and Isocitric acid), fatty acid metabolism (with up-regulated Palmitic acid and L-Palmitoylcarnitine) and tryptophan metabolism (with up-regulated 5-Hydroxy-L-tryptophan, L-Kynurenine, Kynurenic acid, and 5-Hydroxyindoleacetic acid) were significantly associated with frailty phenotype. Conclusions: Our results revealed characteristics of metabolites of frailty in Chinese older adults. The citrate cycle related metabolites (Isocitrate, (s)-Malate, Fumarate and cis-Aconitate), saturated fat (Palmitic acid), unsaturated fatty acid (Arachidonate and Linoleic acid), and some essential amino acid (Tryptophan) might be candidate biomarkers for early diagnosis of frailty. Disorders of energy metabolism, lipotoxicity of saturated fatty acids, disturbances of unsaturated fatty acid metabolism, and increased degradation of tryptophan were potential mechanisms and therapeutic targets of frailty.

